# Correction: Park, B.Y., et al. Is Internet Pornography Causing Sexual Dysfunctions? A Review with Clinical Reports. *Behav. Sci.* 2016, *6*, 17

**DOI:** 10.3390/bs8060055

**Published:** 2018-06-01

**Authors:** Brian Y. Park, Gary Wilson, Jonathan Berger, Matthew Christman, Bryn Reina, Frank Bishop, Warren P. Klam, Andrew P. Doan, Behavioral Sciences Editorial Office

**Affiliations:** 1 Flight Surgeon, Fleet Logistics Support Squadron 40, Norfolk, VA 34800 Bob Wilson Drive, San Diego, CA 92592, USA; brian.y.park4.mil@mail.mil; 2 The Reward Foundation, 5 Rose Street, Edinburgh EH2 2PR, UK; GWilson@rewardfoundation.org; 3 Department of Urology, Naval Medical Center San Diego, 34800 Bob Wilson Drive, San Diego, CA 92592, USA; Jonathan.Berger@med.navy.mil (J.B.); matthew.christman@med.navy.mil (M.C.); 4 Department of Mental Health, Naval Medical Center San Diego, 34800 Bob Wilson Drive, San Diego, CA 92592, USA; bryn.reina@med.navy.mil (B.R.); warren.p.klam.civ@mail.mil (W.P.K.); andrew.p.doan.mil@mail.mil (A.P.D.); 5 Department of Ophthalmology, Naval Medical Center San Diego, 34800 Bob Wilson Drive, San Diego, CA 92592, USA; frank.bishop@med.navy.mil; 6 MDPI, St. Alban-Anlage 66, 4052 Basel, Switzerland

The conflict of interest section of the published paper [1] has been updated as follows:

“Gary Wilson is the author of *Your Brain on Porn: Internet Pornography and the Emerging Science of Addiction.* He holds an unremunerated, honorary position at The Reward Foundation, the Registered Scottish Charity to which his book proceeds are donated. The authors declare no other conflicts of interest. Opinions and points of view expressed are those of the authors’ and do not necessarily reflect the official position or policies of the U.S. NAVY or the Department of Defense.”

In addition, the academic editor’s name has been removed from the manuscript.
